# Anti-inflammatory cellular targets on neutrophils elucidated using a novel cell migration model and confocal microscopy: a clinical supplementation study

**DOI:** 10.1186/s12950-017-0177-0

**Published:** 2018-01-05

**Authors:** T. Smith, L. Engelbrecht, C. Smith

**Affiliations:** 1 Department Physiological Sciences, Science Faculty, Stellenbosch University, Stellenbosch, South Africa; 2 Central Analytical Facility, Stellenbosch University, Stellenbosch, South Africa

**Keywords:** Neutrophil, Chemokinesis, Macrophage phenotype, Grape, Inflammation, ROCK localisation, Human model

## Abstract

**Background:**

In vivo studies have shown grape seed-derived polyphenols (GSP) to benefit in recovery from muscle injury by modulation of neutrophil infiltration into damaged tissue, thereby reducing secondary damage, as well as by facilitating an early anti-inflammatory macrophage phenotype shift. The current study aimed to provide data in this context from human models and to elucidate specific molecular targets of GSP.

Using a placebo-controlled, double-blind study design, eighteen normally healthy volunteers between the ages of 18–35 years old (13 female and 5 male) were orally supplemented with 140 mg/day of GSP for 2 weeks.

Blood samples (days 0 and 14) were comprehensively analysed for in vitro neutrophil chemokinetic capacity towards a chemotaxin (fMLP) using a novel neutrophil migration assay, in combination with live cell tracking, as well as immunostaining for neutrophil polarisation factors (ROCK, PI3K) at migration endpoint. Macrophage phenotype marker expression was assessed using flow cytometry.

**Results:**

fMLP induced significant chemokinesis (*P* < 0.01), validating our model. GSP did not exert a significant effect on neutrophil chemokinesis in this non-compromised population, but tended to decrease overall ROCK expression in fMLP-stimulated neutrophils (*P* = 0.06). Macrophage phenotype markers CD274 and MPO – indicators of a pro-inflammatory M1 phenotype – seemed to be normalised relative to baseline expression levels after GSP treatment.

**Conclusions:**

Current data suggest that GSP may have a modulatory effect on the ROCK-PI3K-PTEN system, but results in this normal population is not conclusive and should be confirmed in a larger, more inflamed population. Potential modulation of macrophage phenotype by GSP should be investigated further.

**Electronic supplementary material:**

The online version of this article (10.1186/s12950-017-0177-0) contains supplementary material, which is available to authorized users.

## Background

Arguably one of the most important and unique aspects of inflammation, is the ability of phagocytic immune cells to migrate through tissue to the site of injury or pathogenic presence. Efficient, directionally accurate movement of phagocytes to a site of injury will result in faster repair by the minimum number of tissue-infiltrating immune cells. This will not only lead to faster resolution of inflammation, but will also result in less secondary damage to otherwise healthy tissue (and thus milder clinical symptoms of inflammation), as approximately 80% of secondary tissue injury has been ascribed to the normally excessive response of neutrophils [1], which includes the release of reactive oxygen species and proteases [2]. Also, in terms of mononuclear phagocytes, macrophage phenotype M1 is recognised as a pro-inflammatory phenotype capable of migration into tissue (thus potentially exacerbating inflammation), while the more anti-inflammatory M2 phenotype seems unable to leave circulation to infiltrate tissue [3–5].

Grape seed-derived products – and polyphenols (GSP) in particular – have received much attention in terms of its beneficial effects as anti-oxidant [6, 7], although it is also known to have anti-inflammatory effects [8, 9]. Since the anti-inflammatory effects of grape-derived products have received relatively less attention in terms of research, despite the well-known links between oxidative stress and inflammation, this was a particular focus of the current study.

Despite several recent reviews highlighting the many benefits to be derived from grape-derived products [9, 10], the majority of data reported was from pre-clinical models. In fact, in terms of human clinical results, data on the anti-inflammatory effects of GSP is extremely limited due to the recognised difficulty of translating natural product benefits from animal models to human models in research [10]. We have previously shown in pre-clinical investigations in rodents, that GSP altered neutrophil migration into injured tissue, thereby limiting secondary damage and the magnitude of the total inflammatory response [7, 11, 12]. A second effect associated with GSP treatment in vivo in rats, was the predominance of the more anti-inflammatory M2 phenotype [12]. Also, in an in vitro model of HIV-associated neuroinflammation, GSP was shown to limit both pro-inflammatory cytokine secretion and chemotaxin-induced human monocytic cell migration across the blood brain-barrier [13]. Together, these data suggest that GSP targets specific cellular sites to beneficially alter the course of inflammation. However, to our knowledge, these effects have not been investigated in an in vivo human model and information on specific molecular targets of GSP remain limited.

Therefore, the purpose of the current study was to expand on available knowledge by conducting an in vivo supplementation study in human subjects. We hypothesised that a 2-week supplementation with GSP would improve the directional accuracy of neutrophils in response to a chemotactic signal, via modulation of proteins involved in the movement of these phagocytes. In addition to our primary aim of investigating the effect of GSP supplementation on cellular role players facilitating neutrophil chemotactic movement, a second aim was the design and implementation of a novel cell migration assay with which to measure both functional outcome (cell migration) and mechanisms (molecular role players) in parallel and in the same cells, so that functional capacity may be correlated with altered expression or activation of cellular targets.

## Methods

### Ethical considerations

Application for registration of the natural product used as medicine at the MCC was still pending at the time of manuscript preparation (ref# 23128). This clinical trial was registered with the South African Clinical Trials Register (NHREC ref. #4242) and clinical trial medical insurance was obtained through Stellenbosch University (ref# PO309814).

Ethical clearance for all protocols was also obtained from the Stellenbosch University Human Research Ethics Committee (ref# M15/09/040) prior to subject recruitment. The study was conducted according to the SA MRC ethical guidelines, guidelines for good clinical practice and the International Declaration of Helsinki. All volunteers signed informed consent prior to entry into the study.

### Subject recruitment

A longitudinal, placebo-controlled, double-blind, human in vivo study design was employed. Normally healthy, non-smoking, recreationally active males and females (*n* = 18, aged 18–25 years), not on any type of supplement, and not suffering from known inflammatory diseases, participated in the study. Subjects were instructed to refrain from taking vitamin, anti-oxidant and anti-inflammatory products and not permitted to perform any strenuous exercise for a period of 14 days prior to initiation of the protocol, as well as for the duration of the entire protocol. A scientist not involved in the study, labelled the placebo and GSP supplements using numerical code. Supplement containers for both treatments were then randomly dispensed to volunteers from one storage bin, as they were recruited. This effectively divided volunteers into two experimental groups, namely GSP supplemented (*n* = 9) and placebo (*n* = 9), ensuring blinding of both participant and investigators involved. All volunteers completed the protocol (refer to Additional file 1 for consort diagram and basic comparative subject demographics). Unblinding was only done at the point of statistical analysis.

### Supplementation protocol and blood collection

A commercially available GSP product (Oxipoven™, Brenn-o-kem Pty Ltd., Tulbach, South Africa) was employed in this study, and consisted of 45% proanthocyanidins, less than 5% monomers and the remainder made up of long chain sugars and glycosides attached to the oligomers, in capsule form. Placebo capsules were prepared by the supplier following standard pharmaceutical procedures as previously described [7], and was identical to the GSP capsules, except for containing no proanthocyanidins. After a 2-week pre-study wash out phase, participants were supplied with GSP or placebo supplements in capsule form, with instructions to consume 2 capsules daily (to equal an intake of 140 mg of proanthocyanidin B in GSP-supplementation group) for a period of 2 weeks. Subjects were required to present their supplements on sample collection days, for confirmation of compliance. On days 0, 7 and 14 blood samples were obtained by venepuncture from a forearm vein, by an experienced phlebotomist. On days 0 (PRE) and 14 (POST), 8 ml heparin blood and 8 ml ethylene diamine tetra-acetic acid (EDTA) blood was obtained from each patient, followed by immediate sample analysis (full blood count, neutrophil migration, immunohistochemistry staining, macrophage phenotyping).

In addition, given the reports of altered adhesion molecule expression after GSP use, as well as a recent report of decreased iron content after mega doses of GSP [14], automated full blood counts and clotting profiles were performed at a commercial pathology laboratory (Pathcare, Stellenbosch, South Africa) after the first 7 days of supplementation, to ensure volunteer safety. (Results were within normal ranges for all participants, with no effect of GSP – Additional file 2).

### Neutrophil migration assay

Neutrophils were isolated from Lithium-heparin blood using density gradient centrifugation with Histopaque 1.077 g/ml (Sigma-Aldrich, #10771) at 2:1 ratio and 6% filtered dextran Cells were resuspended in RPMI and an automated cell count performed (CellDyne 3700Cs, Abbott Diagnostics, Germany), before adjusting cell count to 1 × 10^6^ cells/ml.

**Fig. 1:**
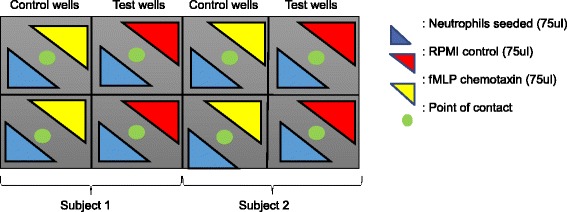
Experimental layout of neutrophil migration assay in 8-well chamber slides

For cell migration, a novel migration assay was developed (Fig. 1). 75ul of isolated neutrophil suspension were seeded to the bottom left hand corner of each well on an 8-well chamber slide (NUNC Lab-Tek II, cat#12–565-8. Then, 75ul of 100μg/ml fMLP (#F3506, Sigma-Aldrich) solution in RPMI 1640 with phenol red (Life Technologies, #12633–020) was added to the top right hand corner of each well. Neutrophil migratory paths were visualized by combining time lapse images acquired every 3 s for 40 min, using 20× magnification on an Olympus Cell system IX-81 inverted fluorescent microscope system with an F-view cooled CDC camera (Soft Imagining Systems) at 37 °C. Image J (Java Software) was used to process the movement data and using the Mtrack J plugin, neutrophil tracks were quantified by measuring the total distance they moved (from starting point to endpoint), as well as the linear distance covered (straight line from start to finish).

### Immunohistochemistry

At the end of image acquisition for live cell tracking, cells were fixed with 4% (*v*/v) paraformaldehyde in PBS for 10 min before permeabilized before blocking with 1% BSA (Bovine Serum Albumin Fraction V, Roche #10735078011) for 20 min. This was followed by immunostaining using appropriate conjugated antibodies (Anti-ROCK1, #AB205432, Anti-PI3Kp110β, #AB202668, both Biocom Biotech) and visualisation (LSM780 confocal microscope with ELYRA S.1 Superresolustion platform (Carl Zeiss, Germany), using ZEN Lite 2012 imaging software). Images were acquired in the desired area of chemotaxis (point for contact of cell suspension with chemotaxin-RPMI or control-RPMI). Mean fluorescence intensity, as well as relative co-localisation of ROCK and PI3K was determined. Co-localization is defined as the presence of two or more different pixels occupying the same space inside the neutrophil. The valid range is between zero and one, with 0 indicative of no co-localization and 1 indicating 100% co-localisation. Values for co-localisation was determined by the relative number of pixels in a channel (PI3K or ROCK) compared to the total number of pixels above the threshold.

### Flow cytometry

Monocytic cells were isolated from EDTA blood using density centrifugation with Histopaque 1.077 g/ml (sigma-Aldrich, #10771) at 2:1 ratio, followed by 42.56% iso-osmotic 1.131 g/ml percoll (Sigma-Aldrich, #E0414) solution in RPMI and phenol red at a 1.25:1 ratio. Pellets of isolated cells were resuspended in Cytofix/Cytoperm Fixation/Permeabilization Solution Kit (#554714, BD Biosience), before phenotyping using flow cytometry and appropriate antibodies (APC anti-CD86, PE anti-myeloperoxidase (MPO), PE-Cy7 anti-CD274, SPC-Cy7, CE anti-HLA-DR, PerCp-Cy5.5 anti-CD163 and BB515 anti-CD206, FITC anti-CD66b, PerCP-Cy5.5 anti-CD106 (VCAM), and BV421 anti-CD54 (ICAM), all from BD Bioscience).

### Statistical analysis

Many of the parameters assessed in the current study, have not been assessed before and thus variability was not known, so that a statistical power estimation could not be performed prior to the study. Subject number was thus chosen following convention in the literature for number of volunteers normally participating in similar human testing. In retrospect, statistical power was poor, with an *n* = 80 calculated as the required number of subjects to return a statistical power of > 80%. This study should thus be considered a pilot study. Statistical analysis was performed using Statistica (version 13.2). Data was analysed for normalcy of distribution, where after main effects of GSP treatment (GSP vs. placebo comparison) was assessed by parametric and non-parametric ANOVA as relevant and post hoc Fischer’s LSD tests. Both absolute values for different parameters at both time points, as well as values for change over time, was compared between treatment groups. All graphical data are presented as means and ± SEM (standard error of means), while tables are presented as means and ± SD (standard deviations), to follow convention for these parameters in the literature. A *p*-value of < 0.05 was considered statistically significant.

## Results

### Neutrophil migration

**Fig. 2:**
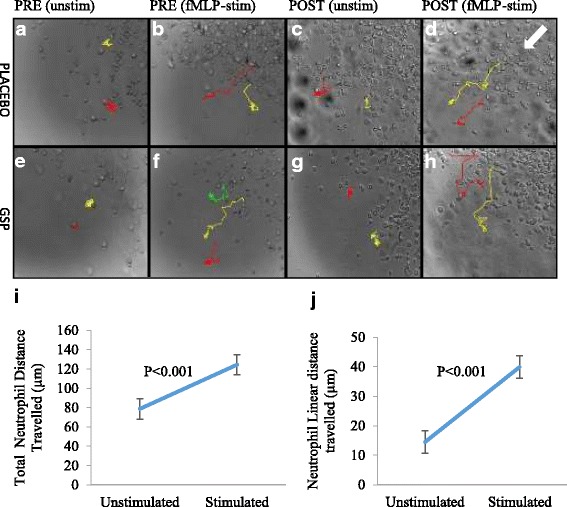
Representative images (**a**-**h**) of placebo and GSP-treated neutrophil chemokinesis and pooled (main effects ANOVA) quantitative results (**i**-**j**) demonstrates the significant effect of fMLP-stimulation, irrespective of treatment, on chemokinetic movement of neutrophils. The migration pathway tracked for the neutrophils is seen by the coloured lines on each image. The white arrow indicate the expected/ideal direction of movement towards the chemoattractant. Images were acquired at 200× magnification (*n* = 9 per group, for all conditions and time point groups, as presented in Table 1). Numerical data are expressed as means and standard error of means (SEM). Statistical analysis: ANOVA and post hoc Fischer’s LSD test

Representative images are presented for the placebo- and GSP-treated groups (Fig. 2a-h). Although no difference was observed over time or between treatment groups, there was a clear increase in cell migration under fMLP-stimulated conditions, validating our model in terms of sensitivity to the chemotaxin. Numerical data supports this interpretation (Fig. 2i-j), indicating that fMLP stimulation significantly increased both the total and linear (ANOVA main effect of stimulation, both *P* < 0.05) neutrophil distance travelled when compared to unstimulated conditions. Group-specific quantitative results again confirmed visual results, with no significant treatment or time effect (Table 1).

**Table 1:** Total and linear distances travelled by fMLP-stimulated neutrophils collected from individuals before and after 2-weeks of placebo or GSP supplementation. Values are expressed as means and standard deviations (SD)

Neutrophil chemokinetic indicators	PLACEBO				GSP			
	Unstimulated		Stimulated		Unstimulated		Stimulated	
	PRE	POST	PRE	POST	PRE	POST	PRE	POST
	Mean (SD)	Mean (SD)	Mean (SD)	Mean (SD)	Mean (SD)	Mean (SD)	Mean (SD)	Mean (SD)
Total Distance (um)	65.08 (37.30)	73.26 (35.46)	83.32* (54.66)	143.21* (60.77)	92.64 (56.14)	81.24 (43.68)	112.90* (69.78)	155.78* (67.11)
Linear distance (um)	12.67 (13.93)	16.61 (10.470	26.66^a^ (16.57)	53.19 (29.16)*	13.54 (10.71)	13.52 (42.91)	35.30^a^ (25.27)	42.91* (29.01)

Statistical analysis: ANOVA and post hoc Fischer’s LSD test, (*n* = 9 per group)

^*^P < 0.05 at least, effect of stimulation; ^a^effect of stimulation, *P* = 0.06

No effect of time or treatment

### Molecular role players facilitating neutrophil migration

**Fig. 3:**
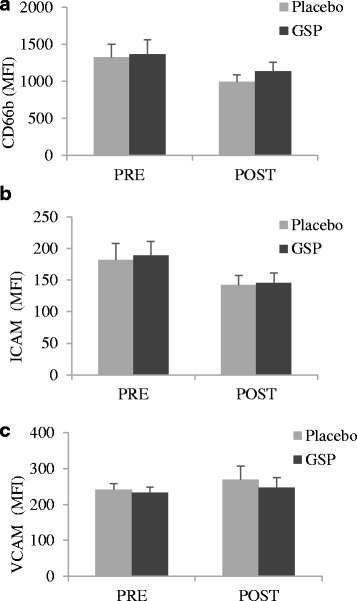
Effect of 2-week in vivo placebo and GSP supplementation on neutrophil protein expression: (**a**) CD66b, (**b**) ICAM-1 and (**c**) VCAM-1. Data are expressed as means and standard error of means. (*n* = 9 per group)

GSP supplementation had no apparent effect on neutrophil surface expression of CD66b, ICAM-1 or VCAM-1 (Fig. 3a-c). Representative flow cytometry scatter plots and fluorescence graphs are presented in Additional file 3.

**Fig. 4:**
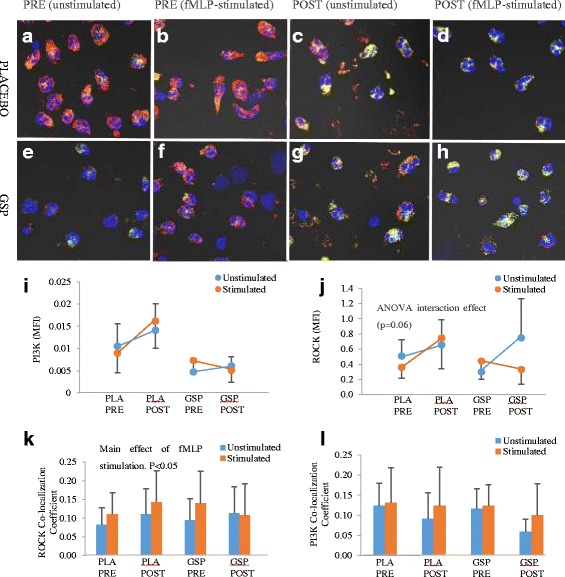
Effect of GSP treatment on neutrophil ROCK and PI3K expression (*n* = 9 per group). Representative images are presented in (**a**-**h**), with ROCK stained green and PI3K red. Yellow is indicative of co-localization. DAPI (1:20 dilution) stained neutrophil nuclei blue. Images were taken at 200× magnification. Numerical data for ROCK (**i**) and PI3K (**j**) expression, as well as ROCK co-localisation with PI3K (**k**) and PI3K co-localisation with ROCK (**l**) are expressed as means and standard error of means (SEM). Statistical analysis: ANOVA and post hoc Fischer’s LSD test

In terms of intracellular role players, although there is no clear evidence of a treatment effect from qualitative data, both ROCK and PI3K seemed to occur diffusely through in the cell in the absence of a stimulus, with relatively more localised expression and increased co-localisation of ROCK and PI3K under stimulated conditions (Fig. 4a-h). Quantitative data showed that both ROCK and PI3K expression were highly variable between individuals, as well as over time (Fig. 4i-j).

From current pilot data, no statistically significant effect of GSP on expression levels of either ROCK or PI3K was evident, although there was a tendency for an interaction effect (*P* = 0.06) of treatment, time and fMLP stimulation for ROCK specifically (Fig. 4j), suggesting a modified ROCK response to fMLP stimulation after GSP treatment. In order to further explore this possibility, co-localisation patterns of ROCK and PI3K expression was analysed. fMLP-stimulation increased the relative portion of ROCK that co-localised with PI3K (ANOVA main effect, *P* < 0.05) – as expected, validating the model even further. Interestingly, for both PRE groups, as well as PLA POST, ROCK co-localisation on average increased by more than 25% (Fig. 4k), while POST GSP ROCK co-localisation did not reflect a similar change on stimulation. Although this result is not conclusive due to the low statistical power in this study, the possibility that ROCK expression levels – and perhaps also its specific locality within the cell – may be a target via which to achieve a modulatory effect in terms of inflammation, warrants further investigation.

### Macrophage phenotype

**Fig. 5:**
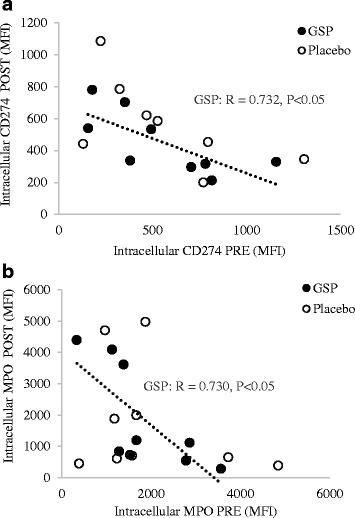
Relationship between monocytic cell (**a**) CD274 and (**b**) MPO expression after in vivo placebo or GSP treatment for 2 weeks. (*n* = 8 placebo, *n* = 9 GSP)

The expression of macrophage phenotype markers in peripheral blood monocytic cells from treatment groups over time are presented in Table 2. Representative flow cytometry scatter plots are provided in Additional file 4. No main effect of treatment or over time was seen, although it should again be noted that both intra- and inter-individual variations were quite large. Interestingly, for CD274 and MPO – both pro-inflammatory indicators – the PRE vs POST values were inversely correlated in the GSP-treated group only (Fig. 5, *P* < 0.05).

**Table 2:** Effects of placebo and GSP treatment on basal expression of macrophage markers in peripheral blood monocytic cells. Data are expressed as means and standard deviation (SD)

Macrophage phenotypic markers	PLA PRE	PLA POST	GSP PRE	GSP POST
	Mean (SD)	Mean (SD)	Mean (SD)	Mean (SD)
CD86 (MFI)	1071 (494)	1345 (469)	1086 (437)	1284 (85)
HLA-DR (MFI)	1145 (602)	1513 (608)	827 (403)	1131 (692)
CD274 (MFI)	601(371)	670 (405)	558 (331)	450 (198)
MPO (MFI)	1940 (1427)	1819 (1813)	1839 (1021)	1869 (1659)
CD206 (MFI)	1896 (1621)	2027 (1204)	1474 (1408)	1440 (953)
CD163 (MFI)	465 (187)	543 (187)	487 (219)	488 (195)
Intracellular IL-10 (MFI)	257 (171)	196 (66)	178 (47)	199 (97)

*Abbreviation: MFI* Median Fluorescent Intensity

Statistical analysis: LSD test (ANOVA), (*n* = 8 placebo, *n* = 9 GSP), no significant difference of time or treatment was evident

## Discussion

Although the data presented is statistically underpowered due to low subject number and high inter- and intra-individual variability, it does provide some insights which may inform on future study design. Current data firstly present a novel method by which in vitro cell migration may be assessed in parallel for both functional capacity and molecular cellular role players, using the exact same cell sample. Secondly, data suggest a role for GSP modulation of cellular role players involved in neutrophil chemotactic movement that is in line with results from animal models and thus warrants further investigation. No effect on migration was observed for GSP treatment. Although several reports of positive effects of polyphenols and GSP on inflammation exist [7, 11, 12] there is relative paucity of information on neutrophil migration and chemokinesis after in vivo GSP supplementation in humans. In contrast to reported in vitro results, current data showed high variability, which may be attributed to different factors, of which at least some are related to study design. Firstly, neutrophils which are isolated and then treated with GSP are exposed to a more controlled environment (in terms of dose of GSP, time of exposure and potential confounding factors), which allows for direct effects of GSP to be detected more readily. In contrast, in in vivo studies, the net effects of GSP is assessed after the supplement has been metabolised – a process which depends on the inherent metabolic rate of each individual assessed. Furthermore, the neutrophil as the first line of defence is arguably the most excitable immune cell, so that it may be activated during the supplementation period for various reasons unrelated to the study intervention. Thus, the relatively larger inter- and intra-individual variations may mask effects.

In order to better understand the variation, a reliability value calculation was performed on both total and linear distance, both between individuals and for the same individuals over time. Briefly, reliability was calculated – the inter-individual repeatability value was 0.61 and 0.43 for total and linear distance respectively, and the intra-individual repeatability value 0.39 and 0.57. This implies that inter-person variation contributed to 61% and 43% of the variability in current data for these two parameters. Statistical power could therefore be improved by addition of more subjects, or increasing the effect size (i.e. using a population more in need of anti-oxidant or anti-inflammatory modulation, such as an aged or inflamed population). A subsequent power analysis indicated that more than 80 subjects would be required to provide sufficient statistical power – a number that is not feasible, given the high cost of analyses. Furthermore, given the fact that day-to-day variation is shown to contribute 39 and 57% of variability for total and linear distance respectively, even data from a bigger group is likely to be complex to interpret in a longitudinal study design. Rather, we recommend that in vivo studies should either be of cross-sectional design – i.e. focus on acute effects on potential molecular targets – or that a very controlled clinical trial should be conducted, during which not only food intake, but also exposure to potential antigens, can be limited. Alternatively, a population with a poorer baseline inflammatory profile may be more ideal to illustrate anti-inflammatory effects, as the expected effect size would be larger. Therefore, although no significant effect was seen in terms of neutrophil movement parameters, given this variability, it does not exclude that GSP may have effects at molecular level.

Since GSP had no significant effect on neutrophil chemokinesis in the healthy population in this study – who probably had limited reserve for improvement of oxidative stress or inflammatory status – these results cannot conclusively inform on effects of GSP on neutrophil migration. Using conventional techniques, i.e. migration assessment in a closed system, the conclusion may have incorrectly been made that GSP had no effect on migration. However, the immunochemistry results obtained from the migrating cells suggest differently, significantly altering the interpretation here.

For example, the consistent aberration in ROCK-related parameters in the GSP-treated group, suggests modulatory effect of GSP on neutrophil cellular parameters in the context of the ROCK-PI3K-PTEN pathway. Neutrophil polarization is a process that causes neutrophils to undergo a mechanical shape change (elongation) which demonstrate a clear leading and tail end of the cell [15]. In terms of the role players assessed in this context, we hypothesised that GSP may have its effect by separating localisation sites of PI3K (to the front) and ROCK (to the rear) in a migrating cell, to improve overall migration capacity and directional accuracy. Futhermore, in terms of the literature on changes in ROCK expression, a decrease has been linked to increased recruitment and migration of neutrophils [16]. In contrast, the loss of ROCK has been associated with neutrophil tail retraction defects [17, 18], suggesting an optimal level of ROCK expression is required for maximum migration capacity. Current data suggests that GSP may modify the ROCK response to fMLP stimulation, although more research is required for a firm conclusion to be made. This is in line with our hypothesis, and may further suggest that GSP may act as such an “optimising modulator” of ROCK. ROCK is known to regulate PTEN – whose role is to dephosphorylate PIP3 to PIP2 and maintain the anterior-posterior PIP3 gradient [19], which is vital for maintaining directional accuracy [20, 21] and its expression level is positively associated with neutrophil migration and the recruitment of neutrophils [22, 23]. Furthermore, current results suggest that a greater extent of co-localization of ROCK with PI3K may occur under stimulated conditions than under unstimulated conditions (≈25%, ANOVA main effect, *P* < 0.05). Interestingly, the same sensitivity was not seen for PI3K. Although the isoform of PI3K assessed in the current study – p110β – has been implicated in cell migration [24], the p110γ isoform has been demonstrated to be more sensitive than the p110β to experimental G protein-coupled receptor mediated activation [25], which may account for the seemingly lower sensitivity of PI3K to ROCK here. This is a limitation of the current study and should be improved on in subsequent trials to ensure optimal sensitivity and relevance. No other literature is available on PI3K and ROCK expression on actively migrating neutrophils, but in light of the increased co-localisation of these parameters after fMLP stimulation, data may suggest increased capacity for cell polarisation after GSP treatment. Importantly, this supports our interpretation that GSP may not necessarily affect total ROCK or PI3K expression levels, but may rather optimise the sites in the cell where they are localised. The decrease in ROCK localisation with PI3K may indeed point towards an improved, GSP-associated polarisation of the cell to have ROCK focused in the rear and PI3K focused in the leading edge of the cell, which should improve migratory economy, as movement will be more directed.

In terms of cell adhesion, plasma levels of soluble adhesion molecules is the more commonly used protocol for assessing cellular adhesion dynamics in the recent literature. An obvious limitation of this approach is that the source of adhesion molecules in plasma cannot be deduced from this, since the shedding of expression markers can be attributed to various cell types. Results in the present study indicated that GSP does not have a significant effect on cellular expression of CD66b, ICAM-1 or VCAM-1. Although one would expect an anti-inflammatory product to exhibit significant modulatory effects on adhesion, our result is in line with the data of Schleimer et al. [26], who similarly reported no change in neutrophil adhesion molecule expression after treatment with IL-4, which is also a known anti-inflammatory modality [27]. In contrast, an in vitro study on the effects of red grape polyphenols on some of these markers [28], reported decreased ICAM-1 protein and gene expression. However, this effect of the polyphenols were only evident after stimulation of the (HUVEC) cells to lipopolysaccharide (LPS), which is a known up-regulator of these adhesion molecules [29–31]. Together, these results suggest that assessment of anti-inflammatory effects under basal conditions is not reflected by cellular adhesion protein expression, and thus that plasma adhesion molecule expression modulation may then reflect modulation of adhesion molecule expression at the level of the endothelium. This in itself is interesting, for two reasons. Firstly, increased lymphocyte adhesion molecule expression has been linked to hypercoagulability and increased risk for atherosclerosis [32]. Secondly, GSP has been reported to decrease VCAM-1 expression [8, 33]. Together, this suggests that while cell adhesion are not compromised – i.e. immune function is maintained in this context – GSP may have additional benefit in the context of atherosclerosis by modification of the endothelium itself. This opens up a new avenue for investigation in the preventative medicine niche.

Finally, our result indicate for the first time in humans, that GSP treatment may indeed exert direct effects on peripheral monocytic cells to limit inflammatory infiltration into tissue, a result we have previously reported in rats with acute skeletal muscle damage-associated inflammation [7, 11, 12] as well as in an in vitro simulation of human HIV-associated inflammatory infiltration across the blood brain barrier [13]. Specifically, the significant inverse correlation between PRE and POST expression levels of both CD274 and MPO within the GSP group only, suggests an adaptogenic role for GSP – i.e. effectively being able to decrease relatively higher M1 protein expression levels while increasing relatively lower levels. These results, reported under *unstimulated* conditions, are especially remarkable in terms of the potential effect of GSP, if one considers that significant changes in M1/M2 markers have only ever been reported after stimulation of cells [34]. Interestingly, type M1 macrophages are known secrete pro-inflammatory cytokines as well as ROS. In excess, ROS can be extremely detrimental to the immune cell itself [35]. Hence, this suggested modulation of M1 phenotype expression by GSP is of importance, as it suggest a potential additional avenue by which GSP may indirectly exert its anti-oxidant effect.

## Conclusions

In conclusion, we provide the first evidence of an immunomodulatory role for GSP in a human in vivo supplementation model, in support of data from animal models. The limited beneficial effects already evident in this pilot study in a healthy, young population – and the absence of any adverse effects – warrants further investigation of GSP via an appropriately powered clinical trial in more compromised populations, such as in aged or chronically inflamed individuals.

## Additional files

Additional file 1: Clinical trial consort diagram and basic comparative subject demographics. (DOCX 34 kb)

Additional file 2: Full blood counts and clotting profiles performed on day 7 of supplementation. (DOCX 14 kb)

Additional file 3: Representative flow cytometry scatter plots and fluorescence graphs for the analysis of CD66b, ICAM-1 and VCAM-1. (DOCX 62 kb)

Additional file 4: Representative flow cytometry scatter plots for the analysis of macrophage phenotype marker expression. (DOCX 203 kb)

## Abbreviations

fMLP: N-Formylmethionyl-leucyl-phenylalanine
GSP: Grape seed-derived polyphenols
HLA-DR: Human leukocyte antigen D
ICAM-1: Intracellular Adhesion Molecule-1
IL-10: Interleukin-10
MFI: Median fluorescent intensity
MPO: Myeloperoxidase
PI3K: Phosphatidylinositol-3 kinase
PTEN: Phosphatase and tensin homolog
ROCK: RhoA-associated coil-containing protein kinase
VCAM-1: Vascular adhesion molecule-1
