# Supplementary material for: Anti-inflammatory cellular targets on neutrophils elucidated using a novel cell migration model and confocal microscopy: a clinical supplementation study
Source: J Inflamm (Lond). 2018 Jan 5;15:2. doi: 10.1186/s12950-017-0177-0 (PMC5756363; doi:10.1186/s12950-017-0177-0)
Supplement: Supplementary file 1 — Clinical trial consort diagram and basic comparative subject demographics. (DOCX 34 kb) [file 12950_2017_177_MOESM1_ESM.docx]

**Additional file 1**

Baseline demographics of recruited subjects (n=9 per group), and consort diagram.

| **Placebo/GSP** | **Gender** | **Age (year)** | **Height (cm)** | **Weight (kg)** | **BMI** |
| --- | --- | --- | --- | --- | --- |
| GSP | Female | 21 | 175 | 65 | 21.22 |
| GSP | Female | 24 | 178 | 62 | 19.57 |
| GSP | Male | 23 | 178 | 81.6 | 25.75 |
| GSP | Female | 22 | 171 | 58 | 19.84 |
| GSP | Female | 29 | 171 | 73 | 24.96 |
| GSP | Female | 21 | 168 | 83 | 29.41 |
| GSP | Male | 19 | 180 | 85 | 26.23 |
| GSP | Female | 21 | 171 | 62 | 21.2 |
| GSP | Male | 28 | 181 | 78 | 23.81 |
| **Mean** |  | **23.1** | **174.8** | **72.0** | **23.6** |
| **SD** |  | **3.37** | **4.68** | **10.39** | **3.33** |
|  |  |  |  |  |  |
| Placebo | Female | 23 | 168 | 65 | 23.03 |
| Placebo | Female | 22 | 150 | 58 | 25.78 |
| Placebo | Female | 21 | 177 | 62 | 19.79 |
| Placebo | Female | 23 | 172 | 55 | 18.81 |
| Placebo | Female | 22 | 165 | 65.5 | 24.06 |
| Placebo | Female | 22 | 157 | 54 | 21.91 |
| Placebo | Male | 21 | 176 | 60 | 19.37 |
| Placebo | Male | 23 | 193 | 100 | 26.85 |
| Placebo | Female | 28 | 163 | 61 | 22.8 |
| **Mean** |  | **22.8** | **169.0** | **64.5** | **22.5** |
| **SD** |  | **2.11** | **12.53** | **13.89** | **2.82** |
|  |  |  |  |  |  |
| **PLA vs. GSP**  **(P-value)** |  | **0.74** | **0.24** | **0.29** | **0.55** |

6 excluded by incl. & excl.criteria

N=0, lost to follow up

N=0, lost to follow up

24 Volunteers

N=18 Volunteers

(Randomised)

Protocol completed with final sample collection and analysis

Protocol completed with final sample collection and analysis

N=9 GSP treated

N=9 Control
