# Supplementary material for: Anti-inflammatory cellular targets on neutrophils elucidated using a novel cell migration model and confocal microscopy: a clinical supplementation study
Source: J Inflamm (Lond). 2018 Jan 5;15:2. doi: 10.1186/s12950-017-0177-0 (PMC5756363; doi:10.1186/s12950-017-0177-0)
Supplement: Supplementary file 2 — Full blood counts and clotting profiles performed on day 7 of supplementation. (DOCX 14 kb) [file 12950_2017_177_MOESM2_ESM.docx]

**Additional file 2**

***Effects of placebo and PCO treatments on full blood count.*** *Blood sample analysis was performed on Day 7 for each participant. Data are expressed as means and standard deviations (SD).*

*Abbreviations: PCO -proanthocyanidolic oligomers, MCV - Mean Corpuscular Volume, MCH - Mean Cell Haemoglobin, MCHC - Mean Corpuscular Haemoglobin Concentration, RDW - Red cell Distribution Width. PT - Prothrombin time, INR - International Normalized Ratio, aPTT - activated clotting time.*

*Statistical analysis: Levene’s test for homogeneity of variance with Games-Howell post hoc test (ANOVA). Placebo n=9 and PCO n=9. No group difference was detected.*

|  |  | **Placebo** | **PCO** |
| --- | --- | --- | --- |
| **Parameter** | **Reference range** | **Mean (SD)** | **Mean (SD)** |
| Total leucocyte count | 4.00 – 11.00 x ${10}^{9}$/L | 6.89 (1.83) | 7.43 (0.90) |
| Neutrophils | 2.00 – 7.50 x ${10}^{9}$/L | 3.83 (1.46) | 4.14 (0.84) |
| Lymphocytes | 1.00 – 4.00 x ${10}^{9}$/L | 2.56 (0.72) | 2.86 (0.38) |
| Monocytes | 0.00 – 0.80 x ${10}^{9}$/L | 0.30 (0.15) | 0.23 (0.14) |
| Eosinophils | 0.00 – 0.40 x ${10}^{9}$/L | 0.18 (0.10) | 0.21 (0.05) |
| Fibrinogen | 2.20 – 5.00 g/L | 3.05 (0.50) | 3.00 (0.41) |
| Platelet count | 140 – 420 x ${10}^{9}$/L | 251 (63.26) | 286.33 (41.65) |
| Total red cell count | 3.70 – 5.30 x ${10}^{12}$/L | 4.92 (0.48) | 4.83 (0.48) |
| Haemoglobin | 11.50 – 15.50 g/dL | 13.74 (1.22) | 13.89 (1.19) |
| Haematocrit | 0.35 – 0.45 L/L | 0.42 (0.03) | 0.42 (0.03) |
| MCV | 81 – 96 fl | 85.44 (3.97) | 87.44 (7.45) |
| MCH | 28 – 35pg | 28.11 (1.62) | 28.78 (3.03) |
| MCHC | 32 – 37 g/dL | 32.78 (0.44) | 32.89 (0.78) |
| RDW | 10 – 15% | 13.30 (1.04) | 12.87 (1.43) |
| PT | 11- 13.5 sec | 12.54 (1.65) | 11.68 (1.19) |
| INR | 0.9 – 1.3 | 1.09 (0.11) | 1.03 (0.05) |
| aPTT | 25.4 – 38.4 sec | 30.10 (2.83) | 30.36 (3.18) |
