# Supplementary material for: Anti-inflammatory cellular targets on neutrophils elucidated using a novel cell migration model and confocal microscopy: a clinical supplementation study
Source: J Inflamm (Lond). 2018 Jan 5;15:2. doi: 10.1186/s12950-017-0177-0 (PMC5756363; doi:10.1186/s12950-017-0177-0)

**Additional file 3**

Representative flow cytometry panels of neutrophils after multiple-staining using anti-CD66b, anti-ICAM and anti-VCAM fluorescent antibodies. Vertical lines indicate fluorescence thresholds while multiple stained populations found on the right side of the thresholds are indicative of positive staining for the fluorescent labels.


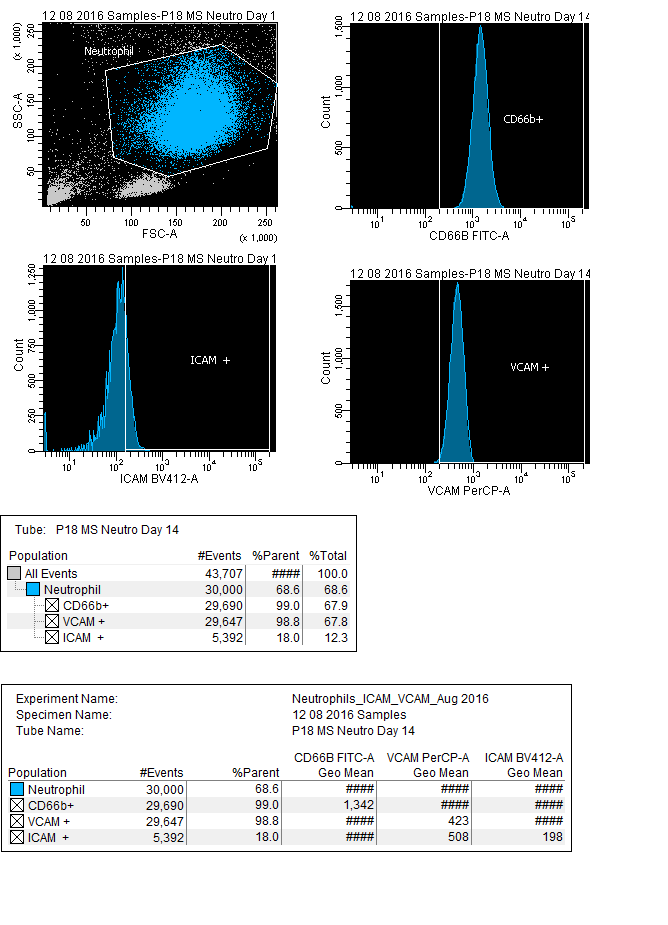

Supplement: Supplementary file 3 — Representative flow cytometry scatter plots and fluorescence graphs for the analysis of CD66b, ICAM-1 and VCAM-1. (DOCX 62 kb) [file 12950_2017_177_MOESM3_ESM.docx]
