# Supplementary material for: Anti-inflammatory cellular targets on neutrophils elucidated using a novel cell migration model and confocal microscopy: a clinical supplementation study
Source: J Inflamm (Lond). 2018 Jan 5;15:2. doi: 10.1186/s12950-017-0177-0 (PMC5756363; doi:10.1186/s12950-017-0177-0)

**Additional file 4**

Representative flow cytometry panels showing a multiple-stained macrophage population, after staining for M1 (CD86, HLA-DR, CD274, MPO) and M2 (CD206, CD163, intracellular IL-10) markers. The data indicates that the markers used are thus expressed and indeed cross the threshold.


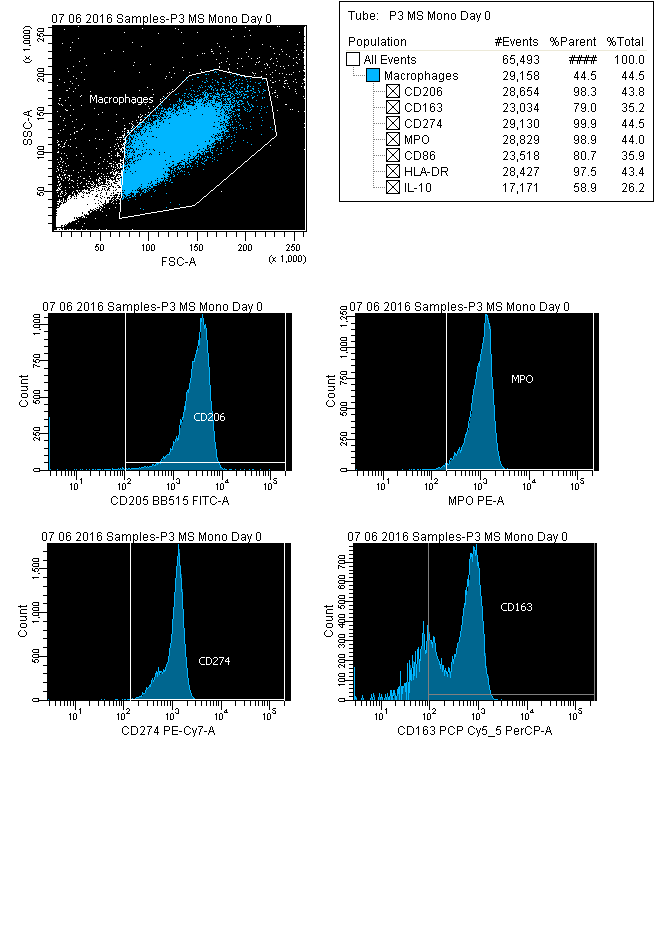

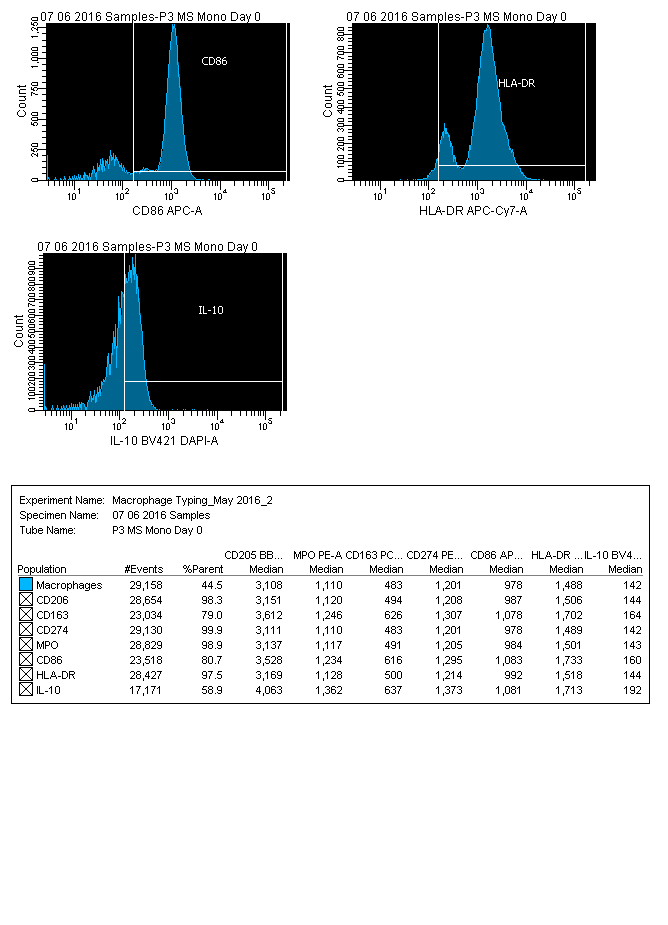

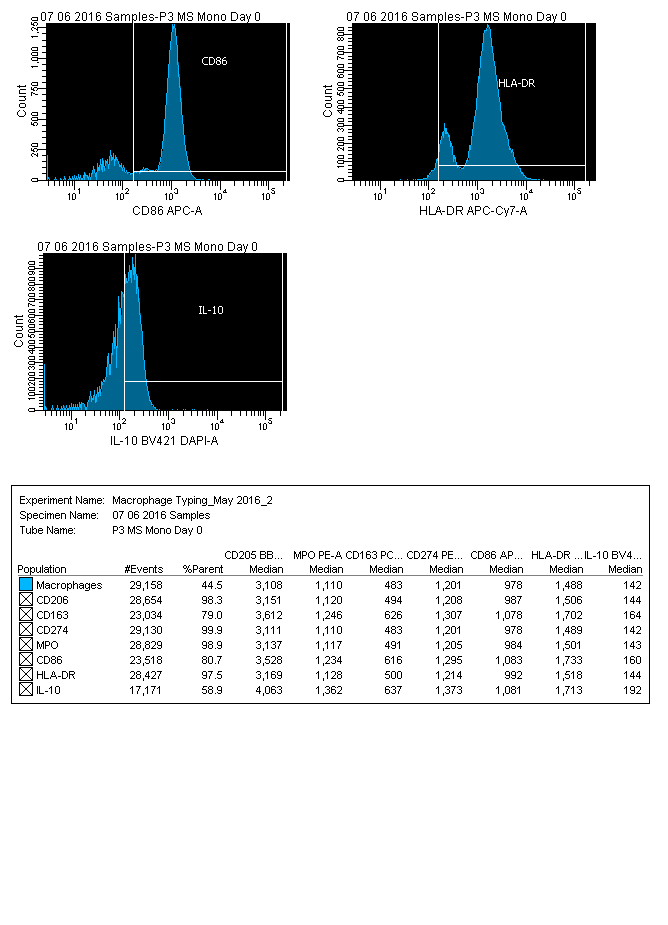

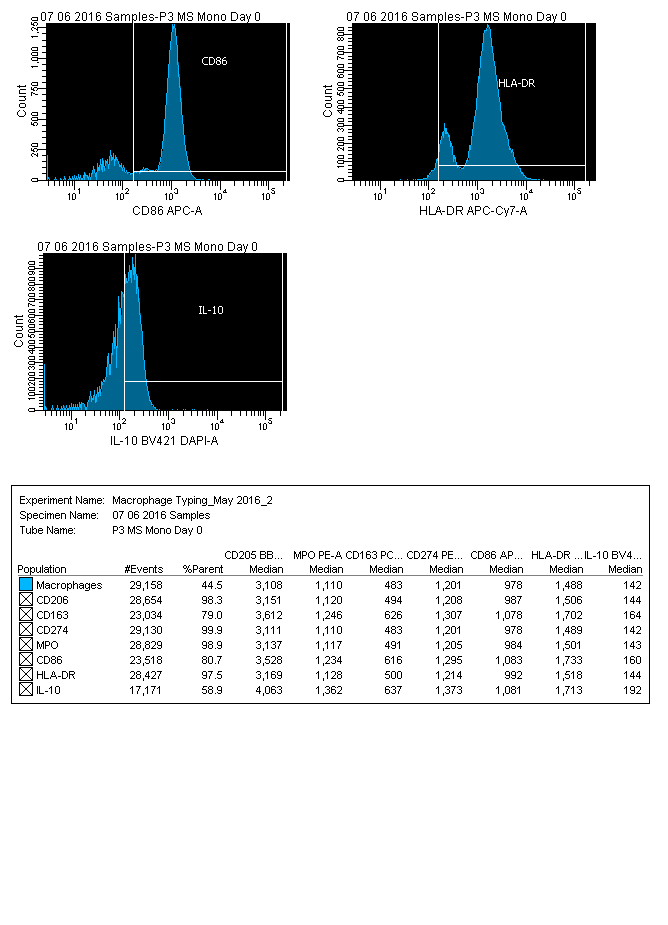

Supplement: Supplementary file 4 — Representative flow cytometry scatter plots for the analysis of macrophage phenotype marker expression. (DOCX 203 kb) [file 12950_2017_177_MOESM4_ESM.docx]
